# An Insight into Anti-Inflammatory Activities and Inflammation Related Diseases of Anthocyanins: A Review of Both In Vivo and In Vitro Investigations

**DOI:** 10.3390/ijms222011076

**Published:** 2021-10-14

**Authors:** Zilong Ma, Bin Du, Jun Li, Yuedong Yang, Fengmei Zhu

**Affiliations:** 1 College of Food Science and Technology, Hebei Agricultural University, Baoding 071001, China; mzl2582@163.com; 2 College of Food Science and Technology, Hebei Normal University of Science and Technology, Qinhuangdao 066004, China; spgcx@163.com; 3 Hebei Key Laboratory of Natural Products Activity Components and Function, Hebei Normal University of Science and Technology, Qinhuangdao 066004, China; dubin@hevttc.edu.cn (B.D.); kycyyd@126.com (Y.Y.)

**Keywords:** anthocyanins, inflammation, NF-κB, MAPKs, inflammation related diseases, in vitro, in vivo

## Abstract

Anthocyanin is a type of flavonoid pigment widely present in fruits and vegetables. It can not only be used as natural pigment, but also has a variety of health functions, for instance, anti-oxidant, anti-inflammatory, anti-tumor, and neuroprotective activities. Persistent proinflammatory status is a major factor in the development, progression, and complications of chronic diseases. Not surprisingly, there are thus many food ingredients that can potentially affect inflammation related diseases and many studies have shown that anthocyanins play an important role in inflammatory pathways. In this paper, the inflammation related diseases (such as, obesity, diabetes, cardiovascular disease, and cancer) of anthocyanins are introduced, and the anti-inflammatory effect of anthocyanins is emphatically introduced. Moreover, the anti-inflammatory mechanism of anthocyanins is elaborated from the aspects of NF-κB, toll like receptor, MAPKs, NO, and ROS and the main efficacy of anthocyanins in inflammation and related diseases is determined. In conclusion, this review aims to get a clear insight into the role of anthocyanins in inflammation related diseases.

## 1. Introduction

Inflammation is a normal physiological response, which is the protective response of the innate immune system to pathogens and injuries, usually temporary, and is one of the body′s oldest defense mechanisms [1]. While acute, localized inflammation is a life-saving mechanism to protect the body from pathogens, repeated stimulation or ineffective regulation can lead to chronic inflammation, damage the body, and induce a variety of diseases [2]. Inflammation is controlled by many factors, and NF-κB signaling pathway is one of the main influencing factors of inflammation. The activation of NF-κB can stimulate the expression of many genes and produce various cytokines, such as TNF-α, IL-6, IL-1β, MCP-1, adipokines, cell adhesion molecules, sVCAM-1 and sICAM-1, and acute phase protein (CRP) [3]. Other important inflammatory factors include PRRS, such as TLRs, and kinases such as MAPK and JNK. When these kinase cascades and nuclear transcription factors are stimulated by external stimuli such as endotoxin, viruses, ROS, cellular redox status, fatty acids, cytokines, growth factors, and carcinogens, inflammation is induced [4].

Chronic, low-grade, systemic proinflammatory state is the risk factor of insulin resistance, metabolic syndrome, atherosclerosis, type II diabetes, cardiovascular disease, and other diseases [5]. Studies have shown that a chronic inflammatory environment is a risk factor for cancer. Chronic inflammation is closely related to tumorigenesis, including cell transformation, promotion, survival, proliferation, invasion, angiogenesis, and metastasis [6]. Atherosclerotic thrombosis is often accompanied by inflammation [7]. Inflammation also regulates the production of acute phase proteins, such as CRP, a subclinical inflammatory marker associated with atherosclerosis. The role of many proinflammatory cytokines in the progression of atherosclerosis has been verified in many studies [8]. Activation of NF-κB signaling pathway produces a large number of pro-inflammatory factors, such as pcam-1, which is involved in the increase of monocyte adhesion and vascular inflammation. TNF-α is related to the pathogenesis of endothelial dysfunction [9]. Many previous studies have shown that inhibition of TNF-α expression can effectively reduce endothelial dysfunction [10,11]. In addition, obesity is closely related to inflammation, which is a manifestation of chronic and low-grade inflammation. Hotamisligil et al. first reported that the fat mass increases with the increase of TNF-α expression [12]. In obesity, the morphological changes of adipocytes lead to the change of secretory response, which is conducive to the inflammatory state. The expanded adipose tissue aggregates macrophages to produce pro-inflammatory proteins such as TNF-α, IL-6, and MCP-1, which further promote the inflammatory state [1]. In conclusion, many diseases are prevented and treated by reducing the inflammatory cascade.

Anthocyanins widely exist in plant cell fluid and are secondary metabolites of plants, such as berries, soybean seed, purple potato, purple cabbage, and black carrot [13]. They have different colors in different environments. Research showed that anthocyanins showed different colors at different pH values, being blue in alkaline environments and purplish red in acid environments. The characteristics of anthocyanins make plants show different colors in different environments. Therefore, anthocyanins of various colors can be produced in the fruits, flowers, and leaves of plants [14]. The change of temperature also has a significant effect on their color [15]. Anthocyanins are widely used as natural pigments because of their brilliant colors. Anthocyanins are a type of polyphenol pigment which belongs to flavonoids, and has many health functions, such as anti-cancer [16], anti-inflammatory [17], neuroprotective [18], eye protection, and so on [19].

**Figure 1:**
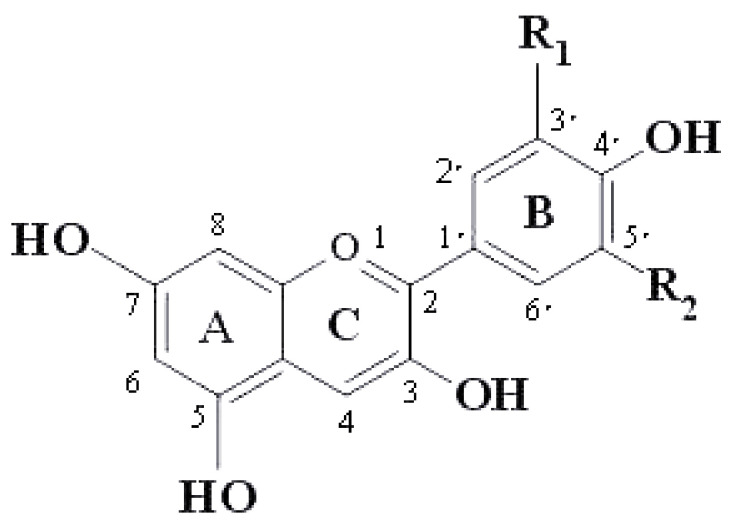
Basic structure of anthocyanins.

Anthocyanins are flavonoid compounds formed by glycosidic bonds between anthocyans and sugars. They are polyhydroxy and methoxy derivatives of 2-benzopyrene or xanthate ions. Their basic structure is C6-C3-C6, namely two aromatic rings and one oxygen-containing heterocycle [20]. Due to the different substituents on the C6-C3-C6 nucleus, the ability to form resonance structure and the environmental factors, various anthocyanins are formed, showing a variety of different colors. At present, there are 22 types of anthocyanins in nature, and 6 types of common anthocyanins in food [21]. The chemical structure of anthocyanins is shown in Figure 1. The types of R1 and R2 substituents are shown in Table 1.

Because of its strong polarity, it was initially thought that anthocyanins could not be directly absorbed by cells into the animal or human circulatory system. However, in vivo bioavailability tests have fully confirmed that anthocyanins can be absorbed through the gastrointestinal tract in prototype form, enter the circulatory system for transfer, transformation, and then excreted through urine [22]. Among them, stomach and small intestine are the main sites of anthocyanin absorption. Under the action of gastric acid, anthocyanins in food can be fully released and dissolved, and most of them can combine with bilirubin translocation enzyme to promote it to pass through the gastric wall mucosa, thus the absorption speed is relatively fast [23]. Studies have shown that the concentration of anthocyanins reached its peak at 2 h after ingestion and disappeared at 4–6 h after ingestion [22]. Anthocyanins in human intake are mainly from berries, vegetables, and other foods. Zamora et al. [24] found that anthocyanin intake was 26.2–90.9 mg/d, through a survey of residents in ten Western European countries. It has been reported that when the anthocyanin intake level reaches 22.3–25.1 mg/d, it can reduce the risk of myocardial infarction and diabetes [25].

In view of the important role of inflammation in various diseases, this paper reviews the therapeutic effect of anthocyanins on inflammatory related diseases, and discusses the potential anti-inflammatory mechanism of anthocyanins from three aspects: Toll like receptor, MAPKs, and NF-κB and oxidative stress. In addition, the antioxidant activity of anthocyanins can effectively eliminate free radicals, reduce the stimulation of inflammation, reduce the secretion of inflammatory factors, inhibit the activation of inflammation related signal pathways, stimulate the production of anti-inflammatory factors, and effectively reduce the inflammatory reaction. Anthocyanins, as a natural pigment, are non-toxic and harmless, and play an anti-inflammatory role in various inflammatory diseases.

## 2. Therapeutic Effect of Anthocyanins on Inflammation Related Diseases

The properties of anthocyans are very unstable, and there are few free anthocyans [26]. Anthocyans generally react with one or more monosaccharides, disaccharides, and trisaccharides through the condensation reaction of 3, 5, 7 carbon hydroxyl groups to form glycosidic bonds in the form of anthocyanins [27]. There are three types of monosaccharides, such as rhamnose, rhamnose and so on. In addition to condensation with sugars, the hydroxyl groups on the core of anthocyanins and the hydroxyl groups connected to glycosides can combine with one or more acylation groups to form acyl anthocyanins [28].

Anthocyanins are widely distributed in higher plants, especially in dark petals, berries, vegetables, potatoes, and cereal seed coats, which make them red, purple, and even black. The amount of anthocyanins in fruits varies greatly, usually in proportion to color, and is affected by light intensity, temperature during growth, altitude, plant hormones, genes, and other factors. The increase of growth temperature will reduce the synthesis of anthocyanins [29]. With the development of anthocyanins extraction technology, a large number of studies have determined the content of anthocyanins in different plants, such as berries (25–495 mg/100 g (fresh weight)) [1], grapes (181.2–716.4 mg/100 g) [30], and pomegranate (>300 mg/100 g) [31], and vegetables, such as red cabbage (113 mg/100 g) [32], black beans (23.1 mg/100 g) [33], eggplant (85.7 mg/100 g), and red onion (23.8–38.8 mg/100 g) [34].

### 2.1. Therapeutic Effect of Anthocyanins on Obesity

#### 2.1.1. In Vivo Study

Anthocyanins extracted from berries are beneficial to weight loss. Research by Diego Luna Vital et al. found that maize rich in ferulic acid and anthocyanins can prevent obesity by regulating TLRs and MAPK signaling pathways, reducing fat production and inflammation, and promoting energy consumption [35]. Some studies have also shown that berry juice and berry powder have no obvious anti-obesity effect, while anthocyanins extracted from berries have obvious anti-obesity effect [36,37]. The reason may be the instability of anthocyanins, and the denaturation in the processing of fruit juice and fruit powder, which affects the function of anthocyanins.

#### 2.1.2. In Vitro Study

Khan Mi et al. found that anthocyanins in Cornus officinalis inhibit lipid accumulation by regulating adipogenesis and lipogenesis related genes and signaling proteins [38].

### 2.2. Therapeutic Effect of Anthocyanins on Diabetes and Cardiovascular Disease

#### 2.2.1. In Vivo Study

Many studies have shown that anthocyanins have an hypoglycemic effect; thus, it has been widely confirmed that anthocyanins can reduce blood glucose concentration. Anthocyanins can regulate the relaxation and contraction of blood vessels by controlling the activity of nitric oxide synthase and potassium channel [39]. Anthocyanins inhibit collagen, hyaluronic acid, and elastin and other important components of the inner wall of blood vessels, thereby protecting collagen, hyaluronic acid, and elastin from being degraded [40]. Anthocyanins can rapidly increase the oxidative function of mitochondria in muscle cells and brown adipose tissue after supplementing energy to increase the body’s energy metabolism rate [41]. They can also inhibit the activity of hydrolytic enzymes that play a key role in the process of carbohydrate digestion, slow down the hydrolysis process of food, and avoid a sharp increase in blood glucose after a meal [42]. Anthocyanins can promote the expression of glucose vector and accelerate glucose consumption. They can be used to prepare diabetes drugs [43]. The Anahita aboonabi study found that anthocyanins supplementation has a positive effect on cardiovascular metabolic risk factors and the inflammatory cascade reaction in a metabolic syndrome population, which may play a role in the prevention or treatment of atherosclerosis [44]. This has been proved by clinical trials. Four weeks of anthocyanin supplementation significantly decreased cardiometabolic risk factors including the average serum fasting blood glucose (FBG) (by 13.3%) and lipid profiles by significant reductions in triglyceride (by 24.9%) and LDL-C (by 33.1%). These results support the hypothesis that anthocyanin supplementation exerts anti-atherogenicity effects by improving cardiometabolic risk factors and reducing thrombogenicity in the MetS population [9]. Reducing the level of alterable atherosclerotic risk factors is an important goal to prevent cardiovascular disease in the metabolic syndrome population. There are established relationships among metabolic syndrome, oxidative stress, chronic inflammation, and cardiovascular disease [45].

#### 2.2.2. In Vitro Study

Daily intake of anthocyanins also significantly improved cardiovascular disease and coronary heart disease [46]. In another study, the authors induced an obese mouse model with a high-fat diet and fed berries containing methylanthocyanins. The results showed that the metabolic damage of the mouse model was significantly improved. Blueberries and Concord grapes (57% and 33% anthocyanins as malvidin, petunidin, or peonidin, respectively) improved the body composition through individual significant effects on energy expenditure and increased activity. Methylated anthocyanins counteract mitochondrial dysfunction associated with metabolic stress by enhancing mitochondrial respiration and eliminating mitochondrial proton gradients (proton leakage) in the adipose tissue. It is proven that methyl anthocyanins can significantly improve the metabolic damage caused by a long-term high calorie diet [47]. Therefore, anthocyanins have good antioxidant and anti-inflammatory effects, which can improve these diseases.

### 2.3. Therapeutic Effect of Anthocyanins on Cancer

#### 2.3.1. In Vivo Study

Anthocyanins in blackberry, raspberry, and other berries can promote the apoptosis of cervical cancer, rectal cancer, hepatocellular carcinoma, prostate cancer, and esophageal cancer in mice [7]. Liu et al. investigated the anticancer activity of the bilberry anthocyanin combo containing macromolecules by modulating the gut microbiome and inhibiting PD-L1. The results showed that bilberry anthocyanins combo improved the proportion of butyrate in feces and increased intratumoral CD8+ T cell infiltrations. The application of the bilberry anthocyanin combo changed the species diversity of gut microbiome decreased by LCP–chitosan and attained the best control of tumor growth in colon cancer [47].

#### 2.3.2. In Vitro Study

In addition, the anticancer and anti-inflammatory effects of anthocyanins have been widely concerned, and scholars at home and abroad have carried out a large number of studies. Feng et al. studied the effect and mechanism of anthocyanin-3-rutin in black raspberry varieties [46]. The results showed that anthocyanin-3-rutin can induce HL-60 cell apoptosis. Anthocyanin-3-rutin treatment also activated p38, MAPK, and JNK reactive oxygen species (ROS) dependence. It activates Bim through the mitochondrial pathway, and the up-regulation of Bim can promote cell apoptosis. Anthocyanins have no cytotoxicity and have potential application prospects in the treatment of leukemia [46]. The anticancer properties and mechanism of anthocyanins are a hot topic.

In conclusion, anthocyanins have a variety of health effects, as shown in Table 2. They can improve metabolic syndrome and obesity, and have anti-cancer, anti-inflammatory, and vision protection effects. They constitute a good natural health care substance.

## 3. Anti-Inflammatory Mechanism of Anthocyanins

### 3.1. Nuclear Factor-κB Pathway (NF-κB)

**Figure 2:**
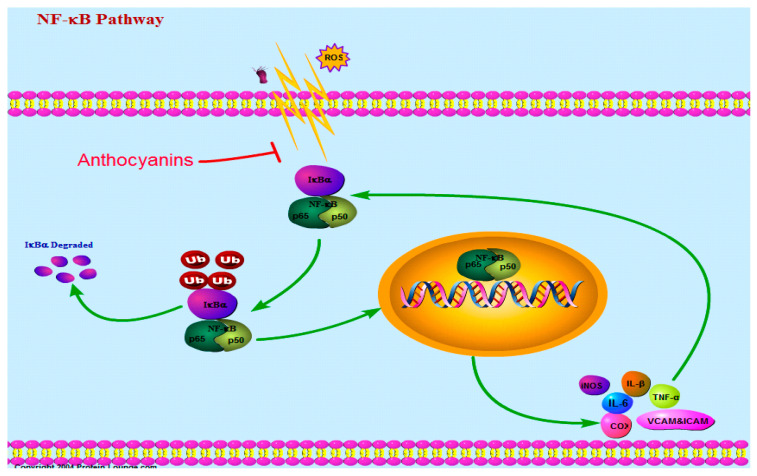
Anthocyanins reduce external stimulation and inhibit the activation of NF-κB signaling pathway. (Ub: ubiquitination).

NF-κB is an important protein complex, which mainly controls DNA transcription and cytokine production. It is a central orchestrator of the inflammatory response [55]. It exists in the cytoplasm of various types of cells in an inactive form. Under normal condition, the binding of p65/p50 heterodimer and its inhibitor protein IκB is inactive. When it is stimulated by external factors, such as ROS, ultraviolet rays, hyperglycemia, and other factors, NF-κB will degrade. The p65/p50 heterodimer is separated from IκB and is in a free state. The free p65/p50 heterodimer transfers to the nucleus and binds to the common DNA sequence to activate the expression of proinflammatory genes, as shown in Figure 2 [56]. Activation of the NF-κB pathway upregulates the expression of proinflammatory cytokines (such as TNF-α, IL-1α, IL-1β and IL-10), chemokines (IL-8), adhesion molecules (ICAMs and VCAM-1), iNOS, COX-2, and cytosolic phospholipase 2 [42].

Duarte et al. found that in LPS induced macrophage model, strawberry anthocyanins significantly inhibited the translocation of p65 subunit from cytoplasm to nucleus, thus inhibiting the activation of NF-κB signaling pathway [57]. Lee et al. (2017) found that the p-Coumaroyl anthocyanins mixture (contains petanin, peonanin, malvanin, and pelanin) extracted from a dark purple-fleshed potato cultivar Jayoung displayed an inhibitory effect on the transcriptional activity and translocation of NF-κB in RAW264.7 macrophages [58]. Another in vitro study reported that a pure sour cherry anthocyanins extract addition to human Caco-2 cells receded the translocation of a p65 subunit from the cytosol to nuclei [59]. Roth et al. treated colon patients with blueberry anthocyanins revealed decreased serum levels of TNF-α, IFN-γ, and activated NF-κB subunit p65 and increased serum levels of IL-10 and IL-22 [60]. IL-10 and IL-22 are anti-inflammatory cytokines involved in wound healing and production of defensins and mucins against bacterial invasion, which can effectively reduce inflammation [61]. Aboonabi et al. found that anthocyanin supplements inhibited NF-κB transactivation and decreased plasma concentrations of pro-inflammatory chemokines, cytokines, and inflammatory mediators and also increased PPAR-γ gene expression. Many studies have shown that anthocyanins inhibit inflammation by inhibiting the activation of NF-κB [62].

### 3.2. TLRs and MAPKs

Toll like receptors (TLRs) are innate immune receptors, which are widely distributed and can recognize the specific structures shared by some pathogens or their products, namely pathogen related molecular patterns (PAMPs) [63]. TLR4 is a receptor that mainly mediates endotoxin reactions, such as LPS, and TLR4/CD14 is an important signaling pathway related to inflammatory response. When the TLR4 receptor binds to a ligand lipopolysaccharide, the protein adapter MyD88 activates the NF-κB signaling pathway, thus promoting the expression of a large number of inflammatory factors, such as TNF-α, IL-6, IL-1β, and COX-2, leading to a persistent inflammatory state [64]. Activator protein 1 (AP-1) is a transcription regulator, which is closely related to the activation of TLR4. AP-1 is assembled through the dimerization of a characteristic bZIP domain (basic region leucine zipper) in the Fos and Jun subunits. Moreover, AP-1 functions are heavily dependent on the specific Fos and Jun subunits, contributing to AP-1 dimers. It is mainly responsible for the control of cell differentiation, proliferation, and apoptosis in the inflammatory state [57]. Both in vivo and in vitro evidence show that anthocyanins can suppress the expression level of COX-2 as well as the transactivation of AP-1, which is a transcription factor that regulates COX-2 gene expression [65,66]. Cui et al. found that in the model of cerebral ischemia–reperfusion injury in mice, feeding *Myrica rubra* anthocyanins can significantly reduce the expression of TLR4 and TNF-α [67]. Anthocyanin pretreatment can also directly regulate ROS level, and the activity of inflammation related downstream pathways, including NO production and SOD activity [68]. In the study of Karunarathne et al., an immunohistochemistry assay revealed that anthocyanins inhibited LPS-induced TLR4 dimerization or expression on the cell surface, which consequently decreased MyD88 recruitment and IRAK4 phosphorylation, resulting in the inhibition of NF-κB activity [69].

MAPKS is also a signal pathway related to inflammation. MAPKs are a family of enzymes that respond to inflammatory stimuli by regulating cell differentiation, mitosis, and apoptosis. MAPK has no catalytic activity in its base form and needs phosphorylation to become active. Three important MAPKs are ERK, which mainly controls cell division, c-JNKs which control transcription, and p38 MAPKs which respond to inflammatory factors. When these MAPKs are activated by inflammatory factors and external environmental factors, they are associated with inflammatory related diseases. The activation of MAPKs directly regulates the activation of AP-1 and synergizes with the NFκB pathway, resulting in gene expression by simulating the promoter gene of many mediators, such as the cytokines IL-6 and TNF-α [70]. Studies have shown that anthocyanins inhibited MLK3 activation and its downstream JNK and p38 MAPK signaling cascades [71]. The protective effect of anthocyanins can be explained by the regulation of oxidative-stress and the suppression of cell apoptosis through the activation of Nrf-2 by interaction with the MAPK and NF-κB signaling pathways [72]. Wongwichai et al. found that anthocyanins and metabolites from purple rice significantly inhibited IκBα degradation, the level of p-p65, and the ERK/MAPK pathway [73]. Many studies have shown that anthocyanins rich plant foods play a protective role through different cell transduction pathways, including inflammatory transcription factors, SAPK/JNK and p38MAPK cascades, JAK/STAT signaling, NF-κB/perk/MAPK, Wnt signaling pathway, and the Nrf2 cell protection pathway [74].

**Figure 3:**
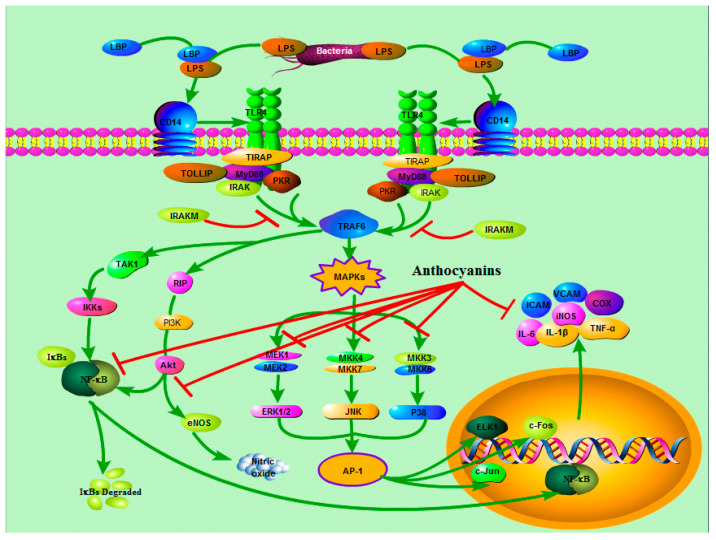
Anthocyanins inhibit TLR4 protein expression and MAPKs signaling pathway. The structure of TLR4 is divided into three domains: extracellular domain, transmembrane domain, and intracellular domain. Extracellular LPS binds to CD14, and since CD14 does not have a transmembrane domain, it binds to the extracellular domain of TLR4 to a transmembrane-mediated endotoxin. When the signal was transferred into the cell, the MyD88 adaptor protein and toll–irak complex began to be recruited. The intracellular TIR region of TLR4 binds to the carboxyl end of MyD88, and the amino terminal of MyD88 binds to IRAK again to activate IRAK (IRAK-M, as a negative regulator, can inhibit the phosphorylation of IRAK and interrupt signal transduction). Activated IRAK reactivates TRAF-6 and further activates the NF-κB, MAPKs, and PI3K-Akt signaling pathways, promoting the secretion of NO and inflammatory factors (Ding et al., 2018; Monica et al., 2016).

In conclusion, as shown in Figure 3, anthocyanins play an anti-inflammatory role by inhibiting TLR4 protein expression and activating MAPKs signaling pathway.

### 3.3. Nitric Oxide (NO)

Nitric oxide (NO) is an important messenger molecule. In pro-oxidative conditions, nitric oxide reacts with O^2−^ to form the peroxynitrite anion ONOO^−^, a highly reactive molecule that damages DNA and lipids and promotes inflammation [75]. NO is mainly produced by nitric oxide synthase (NOS) through a series of oxidation reactions, and its three subtypes are neuronal NOS (nNOS), endothelial NOS (eNOS), and inducible NOS (iNOS) [76]. In activated macrophages, iNOS induces and kills bacteria or tumor cells by producing peroxynitrite. However, iNOS can also be induced in endothelial cells and smooth muscle cells, leading to pathological release of nitric oxide, which is the feature of endothelial dysfunction. A large amount of peroxynitrite production maintains the proinflammatory state, excessive vasoconstriction, and thrombosis. Endothelial nitric oxide synthase (eNOS), conversely, is constitutive and mainly activated by shear stress [77]. Larissa et al. found that strawberry anthocyanins (Pelargonidin-3-O-glucoside) could reduce the concentration of pleural effusion, inhibit the expression of iNOS, and reduce the level of NO in a dose-dependent manner in the mouse model of pleurisy [57].

Moreover, the stimulus–secretion coupling of high glucose-induced the synthesis and the release of NO could interact with VEGF [78]. VEGF robustly activated the PI3K-Akt pathway. Akt, the serine/threonine kinase, can activate eNOS to produce NO, thus promoting inflammation [79]. Huang et al. found that blueberry anthocyanins inhibited eNOS activity and changed NO level by inhibiting Akt expression [80]. Nizamutdinova et al. found that anthocyanins (ANT) extracted from Oryza sativa L stimulate wound healing while suppressing superfluous inflammation by inducing vascular endothelial growth factor (VEGF) production in fibroblasts and keratinocytes [81]. Winter et al. found that in LPS induced BV2 microglial inflammation model, pretreatment with protocatechuic acid can significantly reduce the production of NO [82].

In conclusion, a number of studies have shown that anthocyanins can inhibit the expression and activity of iNOS, thereby reducing the harmful pro-inflammatory effect of excessive production of nitric oxide under oxidative stress.

### 3.4. Reactive Oxygen (ROS)

Oxidative stress has been implicated in the damage of various cellular portions involving lipids, proteins, and nucleic acids through oxidation by ROS such as H_2_O_2_, OH^−^, and superoxide anion radical (O^2−^) [83]. The oxidative process involves the pathogenesis of many diseases. In particular, ROS produced by cell redox disorder is involved in the pathogenesis of various inflammatory diseases including skin injury [84]. In the process of inflammation, the cells involved in the inflammatory process are recruited to the injured site, absorb oxygen, and release ROS. In addition, cytokines and chemokines secreted by inflammatory cells can further stimulate inflammatory cells and produce more ROS. As a result, NF-κB and AP-1 are activated, leading to an increased secretion of cytokines. The vicious cycle will aggravate the inflammatory transition and lead to various chronic diseases [1].

As we all know, anthocyanins have good antioxidant activity, which can remove the excess oxidation free radicals in human body. Huang et al. (2018) found that blueberry anthocyanins can significantly inhibit the increase of ROS in endothelial cells induced by high glucose [80]. At the same time, they can inhibit the decrease of antioxidant enzymes activity. Mallow anthocyanins (Mv), the main component of blueberry extracts, can down regulate the expression of Nox4, which is the main catalytic component of NADPH oxidase and an important source of ROS. Mv inhibited the expression of the NOX4 protein by 45.96%. It is proved that blueberry anthocyanins have a good antioxidant effect. Palungwachira et al. found that the intracellular levels of ROS control the level of phosphor-IκBα by activating a kinase or inactivating a phosphatase that is specific to this protein [84]. Therefore, anthocyanins mediated low levels of ROS, by suppressing IκBα phosphorylation, may abolish the specific proteolysis of phosphorylated IκBα that induces NF-κB activation. González-Reyes et al. have found that anthocyanins have a good antioxidation effect in AD models and can significantly reduce the production of ROS [85]. This was also confirmed in studies by Ma et al. [86], anthocyanin-rich berry extracts reduced H_2_O_2_-induced ROS production and LPS induced No production in BV-2 microglia. Ryo Furuuchi et al., through the study of the diet induced obesity mice model, found that taking borsenberry polyphenols and anthocyanins can inhibit the production of ROS in the aorta [87]. In conclusion, the antioxidant activity of anthocyanins can effectively reduce the inflammatory reaction.

### 3.5. Prostaglandin E2 (PGE2)

Prostaglandin E2 (PGE2) is an important physiologically active lipid, mainly derived from membrane phospholipids. Arachidonic acid (AA) is released by phospholipase A2 (PLA2) from membrane phospholipids, and PGE is synthesized from AA via cyclooxygenase (COX-1 and COX-2) and PGE synthase [88]. PGE2 is the most abundant prostaglandin detected in various tissues. It plays a variety of physiological and pathological roles through the expression of 4 PGE receptor subtypes (EP1-4) on the cell surface. Prostaglandin E2 is produced in large quantities in inflammatory areas. PGE2 induces mast cell activation through EP3 receptor signaling pathway, thus enhancing vascular permeability and leading to acute inflammation. PGE2 also promotes Th1 cell differentiation, Th17 cell proliferation, and Th22 cell production of IL-22 through EP2 and EP4 receptors in vitro. In most cases, PGE2 aggravates chronic inflammation and various autoimmune diseases mainly through EP4 receptor.

PGE2 is mainly synthesized by COX with AA as substrate. Therefore, non-steroidal anti-inflammatory drugs, such as aspirin, play a strong anti-inflammatory effect mainly by inhibiting COX activity [89]. As mentioned above, the activation of NF-κB signaling pathway leads to the increase of COX-2 expression, while anthocyanins can inhibit the activation of NF-κB signaling pathway and COX activity, reduce the production of PGE2 and play an anti-inflammatory effect. He et al. found that anthocyanins can reduce the inflammation induced by UVB radiation by scavenging ROS and inhibiting the expression of COX-2 [90]. Park et al. fed asthmatic mice with anthocyanins, and found that anthocyanins can reduce the development of asthma by down regulating Th2 cytokines, proinflammatory cytokines, and COX-2 [91]. Van de Velde et al. extracted anthocyanins from strawberry and blackberry, and found that the extract had 20% inhibitory effect on COX-2 gene expression by LPS stimulated raw 264.7 macrophage model, and the results showed that the anti-inflammatory effect of anthocyanins was particularly significant [92]. In addition, studies have shown that PGE2-EP3 can also activate PI3K signal, and the inhibition of PI3K can significantly inhibit the production of IL-6 induced by PGE2. Ali et al. demonstrated that anthocyanins can regulate PI3K Akt signaling pathway in AD mice [93]. Zhao et al. found that anthocyanins could down regulate the expression of PI3K protein and inhibit the expression and phosphorylation of Akt [94]. In conclusion, a number of studies have shown that anthocyanins inhibit COX activity, reduce PGE2 production, and reduce inflammation by inhibiting NF-κB and PI3K Akt signaling pathways. The mechanisms of action of anthocyanins are as shown. The underlying molecular mechanisms of action of anthocyanins are listed in Table 3.

## 4. Conclusions

Anthocyanins are naturally harmless and play a role in many diseases. Anthocyanins can regulate the vasodilation and contraction of blood vessels by controlling the activity of nitric oxide synthase and potassium channels, inhibit the degradation of important components of the inner wall of blood vessels, and improve cardiovascular diseases. Anthocyanins rapidly increase the oxidative function of mitochondria in muscle cells and brown adipose tissue after supplementing energy to increase the body’s energy metabolism rate, which can prevent obesity. Anthocyanins can also inhibit the activity of carbon hydrolase and exert the effect of reducing blood glucose. In addition, anthocyanins can also activate Bim through the mitochondrial pathway, promote cell apoptosis, and exert certain anti-cancer effects. As a body’s self-defense mechanism, inflammation plays a very complicated role in various diseases. This article reviews the role of anthocyanins in inflammation. Anthocyanins exert anti-inflammatory effects by inhibiting the release of pro-inflammatory factors, reducing the expression of TLR4, and inhibiting the activation of the NF-κB pathway and MAPKs signaling pathway. Meanwhile, they reduce the production of NO, ROS, and PGE2 and avoid repeated stimulation. However, the stability and utilization of anthocyanins are poor, which limits the application of these compounds. Therefore, future research should focus on improving the stability and bioavailability of anthocyanins, so as to better use the anti-inflammatory effect of anthocyanins.

## Figures and Tables

**Table 1:** R1 and R2 Substituents of Six Basic Anthocyanins.

Anthocyanins	R1	R2
Pelargonidin	H	H
Cyanidin	OH	H
Delphinidin	OH	OH
Peonodin	OCH_3_	H
Petunidin	OCH_3_	OH
Malvidin	OCH_3_	OCH_3_

**Table 2:** Health Effects of Anthocyanins.

Scheme	Dose and Duration of the Intervention	Participants	Study Design	Health Effects	References
Wild Norwegian bilberries and blackcurrant	Two capsules twice a day 4 weeks	35 male and female subjects (MetS + healthy) age = 25–75	Randomized, control design Intervention group (*n* = 20)-two capsule twice a day Control group (*n* = 15)-two capsule twice a day	Lowering inflammation and improving glucose and lipid metabolism	[42]
Fruit juice (Apples, strawberries, blueberries, grapes)	750 mL fruit juice taken in three equal portions 55 days	62 healthy male volunteers age = 20–50	Randomized, control design Intervention group (*n* = 30)-750 mL fruit juice is taken in three equal portions Control group (*n* = 27)–750 mL placebo is taken in three equal portions	Improve DNA integrity and might influence lipid metabolism in humans	[46]
Blueberries	150 g or 75 g fresh blueberries per day 21 days	115 male and female subjects (MetS) age = 50–75	A double-blind, placebo-controlled, parallel study	Improved endothelial Function, Improving metabolic syndrome	[47]
Tart cherry juice	240 mL of tart cherry juice twice a day 2 weeks	11 healthy male or female subjects with chronic insomnia age ≥ 50	A randomized, double-blind, placebo controlled clinical trial	improving insomnia	[48]
Fresh ripe berries of cornelian cherry	total anthocyanin 320 mg/d 12 weeks	80 patients with NAFLD age = 25–65	A double-blind randomized clinical trial	Improving NAFLD	[49]
Blood orange juice	50 mg anthocyanins/d and 500 mL blonde orange juice 4 weeks	41 participants (aged 25–84) with a waist circumference > 94 cm (men) and > 80 cm (women)	A randomized controlled trial	Lowering cholesterol	[50]
Black currant	Black currant anthocyanins 50 mg/d 2 years	38 patients with OAG	A randomized, placebo-controlled, double-masked trial	Increase eye blood flow and improve glaucoma	[51]
Black currant	Black currant capsules 300 mg	11 male patients with Parkinson’s disease	Plasma and cerebrospinal fluid were collected from 11 male patients before and after 28 day supplementation of black currant capsules.	Treat neurological conditions with IGF-1 deficiency.	[52]
Bilberry and black currant	Purified anthocyanin 320 mg/d 12 weeks	21 patients with NAFLD	A randomized, double-blind, placebo-controlled pilot trial	Improving NAFLD	[53]
Black soybeans	anthocyanin-rich black soybean testa extracts 2.5 g/d 8 weeks	63 participants defined as overweight or obese by their body mass index (BMI > 23) or waist circumference (WC > 90 cm for males, >85 cm for females)	A randomized, double-blinded, and placebo-controlled clinical trial	Improve blood lipid status, Prevention of abdominal obesity caused by high fiber and low cholesterol diet	[54]

**Table 3:** The Mechanisms of Action of Anthocyanins.

Source of Anthocyanins	Major Anthocyanins and Dose	Model	Biological Effects	References
Strawberry	Pelargonidin-3-O-glucoside Dose: 100–400 mg/kg	Mouse model of pleurisy	Decreased: ADA and MPO Inhibited: IkB-α, JNKMAPK	[57]
Sour cherry	cyanidin-3-rutinoside, cyanidin-3-O-glucoside, and cyanidin-3-O-glucosyl-rutinoside Dose: 50 μg/mL	HUVECs were treated with 100 ng/mL LPS	Decreased: ROS, TNF-α, IL-6, tPA, PGI2, COX-2	[95]
Mahaleb Cherry	Cyanidin 3-(6-(rhamnosyl)glucoside), Cyanidin 3-glucoside, Cyanidin 3-(6-(rhamnosyl)-2-(xylosyl)glucoside), Cyanidin 3-(2-(xylosyl)glucoside) Dose: 60 µg/mL, 50 μg/mL	TEAC, ORAC and model of vascular inflammation	Decreased: ROS, VCAM-1 and ICAM-1	[17]
Black currant	Delphinidin 3-(6-(rhamnosyl)glucoside), Cyanidin 3-(6-(rhamnosyl)glucoside) Dose: 60 µg/mL, 50 μg/mL	TEAC, ORAC and model of vascular inflammation	Decreased: ROS, VCAM-1 and ICAM-1	
Black Carrot	Cyanidin 3-(6-(6-(feruloyl)glucosyl)-2-(xylosyl)galactoside), Cyanidin 3-(6-(6-(sinapoyl)glucosyl)-2-(xylosyl)galactoside)	TEAC, ORAC and model of vascular inflammation	Decreased: ROS, VCAM-1 and ICAM-1	
“Sun Black” T omato	Petunidin 3-(6-(4-(E-p-coumaroyl)rhamnosyl)glucoside)-5-glucoside (petanin), Malvidin 3-(6-(4-(E-p-coumaroyl)rhamnosyl)glucoside)-5-glucoside Dose: 60 µg/mL, 50 μg/mL	TEAC, ORAC and model of vascular inflammation	Decreased: ROS, VCAM-1 and ICAM-1	
Blueberries	malvidin, malvidin-3-glucoside, malvidin-3-galactoside Dose: 10 μg/mL	HRCECs	Decreased: ROS, VEGF, ICAM-1 Inhibited: Akt, NF-κB Increased: CAT, SOD	[80]
Portuguese blueberries	malvidin-3-galactoside, petunidin-3-arabinoside Dose: 100 mg/kg	TNBS induced colitis in rats	Decreased: iNOS, COX2, MPO, GPX	[96]
Black currant	delphinidin-3-rutinoside, cyanidin-3-rutinoside, delphinidin-3-glucoside Dose: 50 μg/mL	RAW 264.7 macrophages and human THP-1 monocytes	Decreased: IL-1β, iNOS, CXCL9, TNFα Increased: ARG1, CHIL3	[97]
Raspberries	Cyanidin-3-O-sophoroside, Cyanidin-3-O-glucosylrutinoside, Cyanidin-3-O-glucoside, Cyanidin-3-O-rutinoside Dose: 125 μg/mL	HL-60-Human Caucasian promyelocytic leukemia, J45.01-Human acute T cell leukemia	Decreased: LOX, COX-2	[98]
Black rice	cyanidin-3-O-glucoside, peonidin-3-O-glucoside Dose: 25 μg/mL	Rat primary dermal fibroblasts	Decreased: NF-κB p50 and p65 mRNA Increased: Induce Collagen, Type I Alpha 2 mRNA	[84]
Purple rice	Cyanidin-3-O-glucoside, peonidin-3-O-glucoside Dose: 50 μg/mL	Porcine cartilage explant	Decreased: s-GAG, HA, MMP-1, 3 and 13, Inhibited: NF-κB, ERK	[73]
Purple maize	Cyanidin-3-O-glucoside, pelargonidin-3-O-glucoside, peonidin-3-O-glucoside	RAW264.7 macrophages, 3T3-L1 adipocytes	Decreased: PGE2, NO, MCP, iNOS, COX-2, ROS Inhibited: PPARγ, DPP-IV	[99]

## Abbreviations

Nuclear factor-kappa B	NF-κB
Tumor necrosis factor-α	TNF-α
Interleukin-6	IL-6
Interleukin-1β	IL-1β
Monocyte chemoattractant protein-1	MCP-1
soluble vascular cell adhesion molecule-1	sICAM-1
C-reactive protein	CRP
Pattern recognition receptors	PRRS
Toll like receptors	TLRs
Mitogen activated protein kinases	MAPK
c-Jun N-terminal kinases	JNK
Reactive oxygen species	ROS
Cyclooxygenase-2	COX-2
Vascular endothelial growth factor	VEGF
Inducible nitric oxide synthase	iNOS
Hydrogen peroxide	H_2_O_2_
Hydroxyl radical	OH^−^
Nicotinamide adenine dinucleotide phosphate	NADPH
Alzheimer’s disease	AD
Lipopolysaccharide binding protein	LBP
Lipopolysaccharide	LPS
Myeloid differentiation factor 88	MyD88
IL-1 receptor associated Kinase	IRAK
TNF-receptor association factor 6	TRAF-6
TGF-activated kinase 1	TAK1
Toll-interacting protein	TOLLIP
TIR	Toll/IL-1 receptor
RNA-activated protein kinase	PKR
Tissue-type plasminogen activator	tP A
Prostacyclin	PGI2
Human retinal capillary endothelial cells	HRCECs
Catalase	CAT
Superoxide dismutase	SOD
Glutathione peroxidase	GPX
C-X-C motif ligand 9	CXCL9
Arginase	ARG1
Chitinase-like 3	CHIL3
Lipoxygenase	LOX
Sulfated glycosaminoglycan	s-GAG
Hyaluronic acid	HA
Matrix metalloproteinases	MMP
Dipeptidyl peptidase-4	DPP-IV
Proliferator-activated receptor γ	PPARγ
